# Victimization of patients with severe psychiatric disorders: prevalence, risk factors, protective factors and consequences for mental health. A longitudinal study

**DOI:** 10.1186/1471-2458-10-687

**Published:** 2010-11-10

**Authors:** Jack JM Dekker, Jan Theunissen, Rien Van, Jaap Peen, Pim Duurkoop, Martijn Kikkert

**Affiliations:** 1 Arkin Institute for Mental Health, PO Box 75848, 1070 AV Amsterdam, The Netherlands; 2 Professor of Clinical Psychology, Free University of Amsterdam, Room 2B-73, Van der Boechorststraat 1, 1081 CD Amsterdam, The Netherlands; 3 GGZ in Geest: Mental Health Care, Overschiestraat 57, 1062 HN Amsterdam, The Netherlands

## Abstract

**Background:**

Victimization among people with a Severe Mental Illness is a common phenomenon. The objectives of this study proposal are: to delineate the extent and kind of victimization in a representative sample of chronic psychiatric patients; to contribute to the development and validation of a set of instruments registering victimization of psychiatric patients; to determine risk factors and protective factors; and to gain insight into the possible consequences of victimization.

**Methods/Design:**

An extensive data set of 323 patients with Sever Mental Illness (assessed 4 years ago) is used. In 2010 a second measurement will be performed, enabling longitudinal research on the predictors and consequences of victimization.

**Discussion:**

The consequences of (re)victimization have barely been subjected to analysis, partially due to the lack of a comprehensive, conceptual model for victimization. This research project will contribute significantly to the scientific development of the conceptual model of victimization in chronic psychiatric patients.

## Background

Studies conducted in the 1990s found that, in the Netherlands, 75.000 to 100.000 people suffered from chronic psychiatric conditions [1,2]. Changes in mental health care and in the definition of chronic psychiatric conditions have resulted in an increase of 32% in the past decade. In 2009, the national mental health organization GGZ Nederland, calculated that in 2006, 160.000 people suffering from severe long-term psychiatric disorders (0.66% of the Dutch population) received mental health care. Approximately fifty-five to sixty percent of these people were diagnosed with schizophrenia [1,2]. Social reintegration of this group of chronic psychiatric patients, as a result of de-institutionalization, has proved to be troublesome and not without considerable discriminatory and stigmatizing tendencies [3].

In recent decades, studies have shown that psychiatric patients run an elevated risk of victimization [4-6]. The yearly prevalence of victimization among psychiatric patients varies from 16% to 92% [7,8], depending on definitions used, operational modes and sub-populations. In the study by Van Weeghel and Mulder [6], yearly prevalences are internationally estimated between 16% and 60% and nationally between 8% and 20%. Psychiatric patients run a higher risk of victimization than the regular population [4-6]. Theunissen et al. [3] concluded recently that chronic psychiatric patients in ambulatory mental healthcare hardly ever cause serious nuisance. It rather appears to be the other way round, in their case. Patients fall victim to unwanted behavior more often: three-quarters of all patients have experienced discrimination [3]. According to Van Weeghel et al. [6], important risk factors include the severity of the psychiatric symptoms, substance abuse, homelessness, previous victimization, previous perpetration and criminal behavior. Possible risk factors are gender, urban surroundings, disadvantaged areas, ethnicity and the quality of social relations [6].

In this study, we first and foremost wish to determine for a group of severe mental ill psychiatric patients in what way and to what extent these chronic psychiatric patients have fallen victim to violence and/or crime in the recent period, and to what extent they feel safe or secure in their neighborhood and surroundings. For this we will use a cohort of patients we interviewed approximatly 5 years ago. In the period 2005 to 2007, our research group [3] performed the Amsterdam Urban Chronicity Study, with a cross-sectional sample survey among chronic psychiatric patients [9]. A cohort of patients with long-term, serious psychiatric disorders and impairments/limitations/disabilities (Long-term Care Dependent or LCD-patients) was randomly selected (using research criteria for long-term mental illness). The functioning of this cohort was charted (syndrome/clinical profile, symptoms, physical complaints, medication, medication side-effects), as well as the quality of life and the need for care and social integration (size of social network, social support experienced, social nuisance/inconvenience, discrimination). Patient interviews, file data and therapist information were used. Patients in this cohort indicated at that time, not to have any objections to a second measurement/interview.

It has been almost 5 years since this group of patients was interviewed within the framework of this study. At that time, about 170 patients of this group were residing at home and received ambulatory care, about 100 were living in housing facilities for chronic psychiatric patients or in a long-stay unit and about 60 were accommodated in Regional Institute for Sheltered Housing Facilities.

As already stated before, the first objective of this study is to dertemine the extend and type of victimization in this patientgroup. An existing Dutch instrument tested in several epidemiological studies in the population, will be used in this part of the study. Its focus will be on physical, sexual and emotional victimization, experienced discrimination based on the patient's limitation/disability and severity of the consequences of victimization (e.g., physical, emotional, legal consequences).

The second objective of this study is to assess for the second time (after 4 to 5 years) the functioning of the random survey sample of chronic patients of 4 to 5 years ago. We wish to determine (as in the first measurement in the Amsterdam Urban Chronicity Study) the situation of the patient after 5 years of mental health care regarding:

1) symptoms, medication use and its side-effects, their use of psycho-active substances and their additional physical disorders/complaints;

2) quality of life, and how content they are with the care offered;

3) social integration in the city, what are the characteristics and the size of the social networks of patients?

In this way we can determine not only how much and in what way patients were victimized over the past 5 years, but also the relationship of illness characteristics of 5 years ago with the victimization data afterwards (determining risk and protective factors for victimization). Furthermore we were also able to determine the relationship of the victimization and its consequences for the illness characteristics of today.

Thus, this is a retrospective study of victimization incidence rates among chronic psychiatric patients during the past years as established in longitudinal research. By combining these study designs, possible risk factors and protective factors, as well as the possible consequences of victimization will be determined.

## Methods/Design

### Design

This is a longitudinal study conducted among a survey sample of psychiatric patients in mental health care institutions in Amsterdam, who are long-term care dependent. The research population consists of patients with chronic psychiatric problems, as defined by using research criteria for chronicity in the information systems of the mental healthcare institutions 5 years ago.

Patients who participated in the first measurement at that time were informed that follow-up research was possible and were asked to participate. For the second measurement patients will be contacted and asked to participate in this follow-up study, after obtaining verbal or written consent from their therapist. Consenting patients will be invited for an interview, either at their treatment ward/facility or at the patient's home. They will be given information and asked for their informed consent, also for gatering information from other resources. If consent is given, the interview will follow, during which several scales/measures are completed. In addition, information on these patients will be retrieved from care providers or, if necessary, from patient files and mental health care service files (GGD).

### Population, inclusion and exclusion criteria

The target group consists of patients diagnosed as suffering from Severe Mental Illness (SMI) 4 years ago, with or without serious addiction problems. Criteria for SMI were and still are: a serious psychiatric disorder as diagnosed by a psychiatrist (schizophrenia, bipolar disorders, severe/major recurrent depressions) and receiving care for more than 2 years. Most of the interviewed patients (73%) of the original sample showed a global assessment of functioning (GAF) under or equal to 50.

### Feasibility of recruitment and timetable

At the first measurement 4 years ago, we found that patients participated willingly and enjoyed the interview. Patients received a participation compensation of 10 Euros for one interview in advance. At that time, participants in the study were asked if they had any objection to being contacted again for research in years to come. They virtually all indicated that they had no objection. In this study, patients will be asked for two interviews and will be paid 10 Euros per interview. They will be asked to give written consent for access to various resources, like patient files and the crisis center information system as well as information on patients' contacts with the mental health care service (GGD) and the Justice Department. All data will be processed anonymously.

The starting date of this study is scheduled for the end of 2010. During the first twelve months of the research year, all patients of the Amsterdam Urban Chronicity Study will be approached and interviewed, if consent is given. The interviewing will consist of two appointments of 1 to 1.5 hours. Patients will be paid 10 Euros per session. The last 6 months will be used for analysing the data and writing the report.

### Medical ethics issues

The research is in compliance with the Helsinki Declaration, which containes ethical principles for medical research involving human subjects. METiGG, a dutch medical ethics committee for research with patients in psychiatry gave permission 5 years earlier for the first phase of the study. In 2010 permission was established for the second assessment as well, which is in essentian not different from the first.

### Outcome parameters

The victimization instrument will be the Integrale Veiligheidsmonitor or IVM (Integral Safety/Security Monitor). This survey was developed by Statistics Netherlands (CBS), the Dutch Ministry for the Interior and the Justice Department. The IVM is a reliable and valid questionnaire instrument, designed to measure factors including the livability of residential areas, the sense of insecurity/danger and victimization through frequent criminality. Similar to the International Crime Victims Survey (ICVS), people in institutions and residential care homes are not included in the IVM. What makes the IVM special is that municipal councils can voluntarily participate in the study via local oversampling, under strict conditions relating to methodology and questionnaires. Results at (sub)local level are comparable to those at regional and national level. In 2008, 84 municipalities participated, and in 2009 a total of 240. Coordination and organization of the IVM was assigned to the Safety Monitoring Agency (Bureau Veiligheidsmonitor), an independent part of the Nicis Institute (for Urban Research and Practice).

Furthermore, the measuring instruments applied in the first assessment of the Amsterdam Urban Chronicity Study are used, namely:

○ Psychopathology is measured with the Brief Psychiatric Rating Scale-Expanded or BPRS-E [10].

○ Care needs according to patient and therapist are measured with the Camberwell Assessment of Need or CAN [11].

○ Quality of Life is assessed with the Manchester Short Assessment of Quality of Life or MANSA [12].

○ Information on symptoms and disorders and satisfaction with the care provided are assessed with the 'presentation-indicators questionnaire'.

○ The extent to which social support is experienced is measured with the Social Support Questionnaire or SSQ [13].

○ The extent to which discrimination is experienced is measured with the 'Discrimination Scale' a self -developped instrument.

○ Medication adherence is measured with the Brief Adherence Rating Scale or BARS [14].

○ Changes in housing/living, employment and daytime occupation are listed with the help of a questionnaire yet to be developped.

The following instruments are completed by caregivers (e.g., social psychiatric nurses, personal coaches/counselors/attendants, resident doctors/registrar physicians (USA/GB) or psychiatrists):

The extent to which patients are motivated and cooperative in their treatment is assessed with the 'GGZ-Compliance' scale [15].

○ Substance abuse (alcohol and drugs) is rated with the CAGE Questionaire [16].

○ The extent to which patients cause nuisance/inconvenience is measured with the 'Social Nuisance Screening List', also a self-developped instrument.

### Power

With regard to the representativeness and generalizability of our sample of 5 years ago, we make the following observations. In 2005, a total of 4.576 LCD patients were receiving treatment from the Amsterdam GGZ mental health services. Of a non-selective random sample of 876 patients, a total of 323 (37%) were included in the study. This group is representative of the total group of 876 patients on a number of important variables (illness, age, gender). In 2010 we conducted a pilot study to establish how many of the patients from that group could still be reached, 5 years later. We found that 88% (N = 280) were still registered with a GGZ mental health organization or Regional Institute for Sheltered Housing Facilities in 2009. Of a non-selective random sample of 32 patients from this latter group, approximately 15% declined to participate in an interview. The loss-to-follow up for the second measurement will mainly consist of deceased patients and those who have moved away; the dropout rate will therefore be much lower compared to that of the first measurement.

With regard to the overall statistical power of the proposed design: Suppose that, of the 280 patients who are still receiving GGZ treatment, a further 40 refuse to participate, then this will leave a sample of 240 patients. In this group of patients with serious symptoms, often of schizophrenia, a lifetime victimization prevalence of 50% is not unlikely. In that case, with an ES of 0.6 distributed across 2× 120 patients, a two-sided test will yield a statistical power of 90%.

Teplin et al. [17] in Chicago found an annual prevalence of 25% for very serious victimization (robbery, rape, sexual assault, murder) among SMI patients. Based on a 5-year prevalence of 30% (the period between the first and second measurements) a ratio of 74/166, with the same ES of 0.6, will yield 81% power with two-sided testing. This power appears to us to be sufficient to detect differences between victimized and non-victimized patients at baseline and at second measurement.

## Discussion

So far, the consequences of (re)victimization have been subjected to little analysis, partly owing to the lack of a comprehensive, conceptual model for victimization [6]. On the whole, there is little knowledge about the issue of victimization, and care workers are unaware of the size of the problem. According to Van Weeghel et al. [6] few interventions, if any, are available. Moreover, there is no research tradition in the Netherlands when it comes to the victimization of psychiatric patients. In line with this, there are no sufficiently reliable and valid standard measures.

The aim of the proposed study is to expand our knowledge of (1) the degree of victimization in a non-selective group of about 320 chronic psychiatric patients with SMI; (2) the relationship between victimization and discrimination, (self)stigmatizing and perpetration; (3) possible risk factors and consequences of victimization and (4) ideas for prevention programs. The key need is for reliable and valid measuring methods to determine the nature, prevalence, incidence, risk factors and consequences of victimization among patients in different settings, who suffer from SMI: What is the nature, prevalence and incidence within different settings? Furthermore, there are questions concerning the relationship between victimization and SMI, discrimination, (self)stigmatizing and perpetration: What are the consequences of victimization for patients with an SMI? Can an explanatory model be developed?

The research proposed here will allow us to answer most of these questions. On the one hand, through determining victimization with an existing reliable and valid population-based instrument like the IVM, it becomes possible to conduct comparisons both nationally and internationally. On the other hand, by using a longitudinal study among a random survey sample of chronic psychiatric patients, we can enhance our knowledge of possible risk factors and protective factors (like illness characteristics, but also QoL-data and social integration), as well as gaining insight into the possible consequences of victimization, (in the context of developing a theoretical framework for the relationship between victimization and psychiatric disorders).

We trust that our research project will contribute significantly to the scientific development of the conceptual model for victimization in chronic psychiatric patients. In our study, we focus on comprehensive characteristics of psychiatric syndromes, experienced 'quality of life' and individual 'need-for-care', but also on general concepts such as 'social support', size and characteristics of the patient's social network, and the experienced social support. Correlations between these variables and victimization, stigmatizing and perpetration can thus be determined.

## Competing interests

The authors declare that they have no competing interests.

## Authors' contributions

JD, JT and MK were the primary investigator of this study. JD, JT, MK and RV are accountable for the study design and the conceptualization of this study. MK, PD and JP are responsible for the pratical implementation of the study. JP performed the statistical analysis. JD, JT, MK, RV, JP and PD participated in the draft manuscript of the proposal. JD and JT are responsible for the overall supervision of the study. All authors have read and approved the final manuscript.

## Pre-publication history

The pre-publication history for this paper can be accessed here:

http://www.biomedcentral.com/1471-2458/10/687/prepub
